# Paraganglioma of the Tongue in a Chow Chow Dog: A Comparison With the Human Counterpart and Literature Review

**DOI:** 10.3389/fvets.2020.00422

**Published:** 2020-07-24

**Authors:** Fábio Ranyeri Nunes Rodrigues, Jeniffer Mendes da Silva Freire, Luana de Aguiar Paes Fidelis, Alexandra Ariadne Bittencourt Gonçalves Pereira, Davi Emanuel Ribeiro de Sousa, Tais Meziara Wilson, Benito Soto-Blanco, Márcio Botelho de Castro

**Affiliations:** ^1^Veterinary Pathology Laboratory, Campus Darcy Ribeiro, University of Brasília, Brasília, Brazil; ^2^Veterinary Teaching Hospital, Campus Darcy Ribeiro, University of Brasília, Brasília, Brazil; ^3^Department of Veterinary Clinics and Surgery, Veterinary College, Universidade Federal de Minas Gerais, Belo Horizonte, Brazil

**Keywords:** cytology, dogs, immunohistochemistry, neoplasm, paraganglia, oral cavity

## Abstract

Over the last 20 years, substantial knowledge has been developed in Veterinary oncology, and tumors previously reported only in humans have been identified in animals. Primary paragangliomas of the tongue are extremely rare tumors in human beings and have never been reported in animals. A Chow Chow dog showed an ulcerated nodule at the lingual body, deeply infiltrated, which extended to the base of the tongue. A full clinical and pathological investigation was conducted, and a post-surgical follow-up of 6 months did not detect recurrence. Cytological, histological, and immunohistochemical features are presented and support the diagnosis of lingual paraganglioma. The paraganglioma of the tongue reported in this Chow Chow dog shares many similarities with the human counterpart.

## Background

Over the last 20 years, substantial knowledge has been developed in Veterinary oncology. Tumors previously reported only in humans have been identified in animals, and there is a considerable improvement in the diagnosis and treatment of neoplasms and an increase of survival. Neoplasms of the tongue accounted for 54% of lingual lesions, and 64% are malignant tumors, with a particularly high incidence in Chow Chows and Chinese Shar-Peis (1).

Primary paragangliomas of the tongue are extremely rare tumors in human beings and have never been reported in animals. Paragangliomas of the head and neck usually have a parasympathetic origin and generally are non-secretory (2, 3). Extra-adrenal paragangliomas (EPs), also known as chemodectomas, are neuroendocrine tumors derived from paraganglia of the autonomic nervous system (3, 4). In dogs, the most common locations of EPs are mediastinum (aortic body), head and neck (carotid and jugular bodies), and also occur in other infrequent sites such as orbit and abdomen (4–8). EPs have also been reported in orbit (9) and abdomen of horses (10), and in the retroperitoneal and renal region of cats (11, 12).

We describe the occurrence of paraganglioma in the tongue of a Chow Chow dog that shares similarities with the human counterpart.

## Case Description

**Figure 1:**
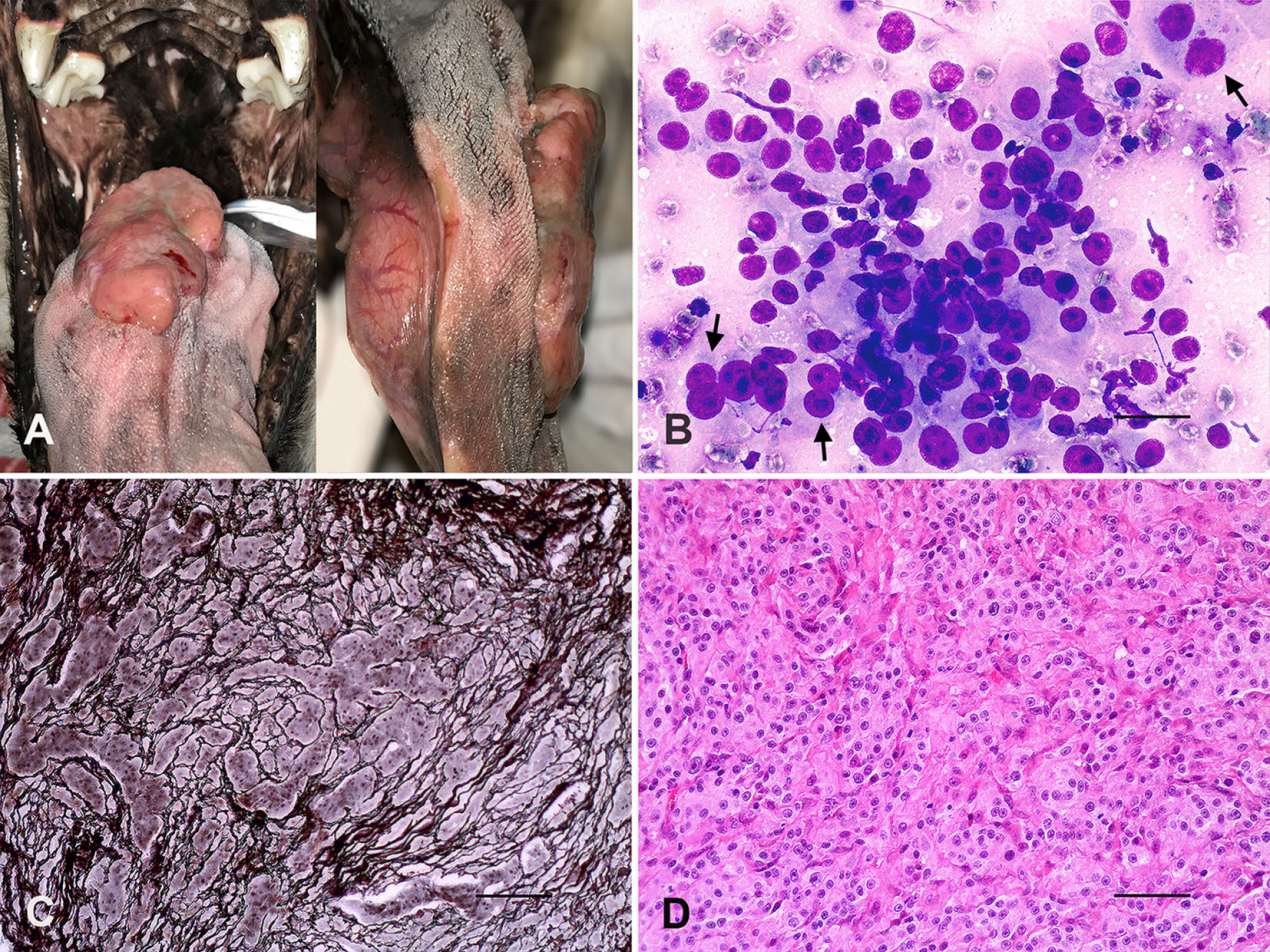
Tongue, paraganglioma. **(A)** Ulcerated exophytic nodule at the right side of the lingual body, which extended to the base of the tongue. **(B)** Round to polygonal cells, moderate anisocytosis and anisokaryosis, nuclei with finely granular chromatin, evident nucleoli, and some naked nuclei and binucleated cells (arrows). Romanovsky stain. Bar = 25 μm. **(C)** Neoplastic cells arranged in rounded nests surrounded by thin fibrovascular trabeculae (“Zellballen” appearance). Reticulin stain. Bar = 100 μm. **(D)** Tumoral cells with rounded nuclei, heterogeneous chromatin, and eosinophilic cytoplasm. H&E. Bar = 50 μm.

An 11-year-old female Chow Chow dog was referred for clinical care with sialorrhea, oral bleeding during eating, fetid breath (halitosis), and dysphagia for 20 days. The inspection of the oral cavity showed a nodule with an irregular surface, exophytic, and ulcerated at the right side of the lingual body, deeply infiltrated, which extended to the base of the tongue (Figure 1A). Regional lymph nodes did not present changes at clinical evaluation, and no other tumoral masses were detected in the oral cavity. Thoracic x-ray, abdominal ultrasonography, and computed tomography (CT) of the head, complete blood cell count, and biochemical assay, pre-surgical fine needle biopsy aspiration of the neoplasm, and a partial glossectomy was indicated.

Samples of tissues were fixed in 10% phosphate-buffered formalin (pH 7.0), embedded in paraffin, and sections of 4 μm stained with hematoxylin and eosin (H&E), periodic acid-Schiff (PAS) and reticulin silver stains. Tumoral samples were also submitted to immunohistochemistry using the biotin-peroxidase-streptavidin method (ImmunoDetector DAB, HRP, BioSB Inc.) with primary antibodies incubated overnight (Table 1). The primary antibodies were omitted on the tissue sections and used as negative controls.

**Table 1:** Antigen, origin of the primary antibodies^♦^, dilutions used in the immunostaining protocols, and immunolabeling of the paraganglioma of tongue in the Chow Chow dog.

**Antigen**	Antibody clone^*^^,^^♦^^,^^§^	**Dilution**	**Positive control^⋆^**	**Immunolabeling**
CrA	DAK– A3	1:500	Pancreas, islets	+
SYN	DAK–SYNAP	1:500	Pancreas, islets	+
S100	Polyclonal	1:400	Brain, astrocytes	+^ρ^
VIM	Vim3B4	1:400	Fibrosarcoma	+
GFAP	Polyclonal	1:400	Brain, astrocytes	+
Melan-A	A103	1:500	Melanoma	–
WS CK	Polyclonal	1:1,000	SCC	–
EMA	E29	1:500	Colon, enterocytes	–

♦ Dako Corporation;

* Antigen retrieval: citrate pH 6.0, 125°C, 3 min, performed in a pressure cooker; ♢ Detection method: biotin–peroxidase–streptavidin;

§ Chromogen: DAB 3,3′-diaminobenzidine;

⋆ Canine tissues;

ρ *sustentacular cells only; SCC, squamous cells carcinoma; CrA, Chromogranin A; SYN, Synaptophysin; S100, S100 protein; VIM, Vimentin; GFAP, glial fibrillary protein; Melan-A, melanoma antigen; WS CK, wide spectrum cytokeratin; EMA, epithelial membrane antigen*.

Additionally, we made a review of manuscripts published on tongue paraganglioma in animals and humans. A PubMed query was conducted with the following keywords: paraganglioma, tongue, pharynx, dog, canine, animals, veterinary, head, and neck. Articles were selected based on the location of paragangliomas and scientific relevance and were used to compare features between the dog and the human counterpart.

CT revealed a poorly delimited neoformation in the body of the tongue, isodense, heterogeneous at the proper lingual, styloglossus, hyoglossus, and genioglossus muscles. Thoracic x-ray and abdominal ultrasonography did not detect tumoral masses or other abnormalities. The pre-surgical evaluation showed an increase in serum activity of creatine phosphokinase (CK 435.7 UI/L, reference range: 69-214 UI/L). The CBC and the serum levels of alanine aminotransferase, aspartate aminotransferase, alkaline phosphatase, γ-glutamyl transferase, creatinine, urea nitrogen, total protein, albumin, cholesterol, triglycerides, calcium, and phosphorus were within the reference ranges (13).

Fine needle biopsy aspiration of the tongue mass revealed round to polygonal cells arranged in groups or isolated, moderate anisocytosis and anisokaryosis, nuclei with finely granular chromatin, some naked nuclei, and the presence of one or two nucleoli. The tumor cells had a slightly basophilic cytoplasm and occasional acinar-like structures, and binucleated cells were also observed (Figure 1B). The partial glossectomy was successful with the complete resection of a solid and firm white-brown nodule of 6.5 × 5.0 × 4.0 cm.

**Figure 2:**
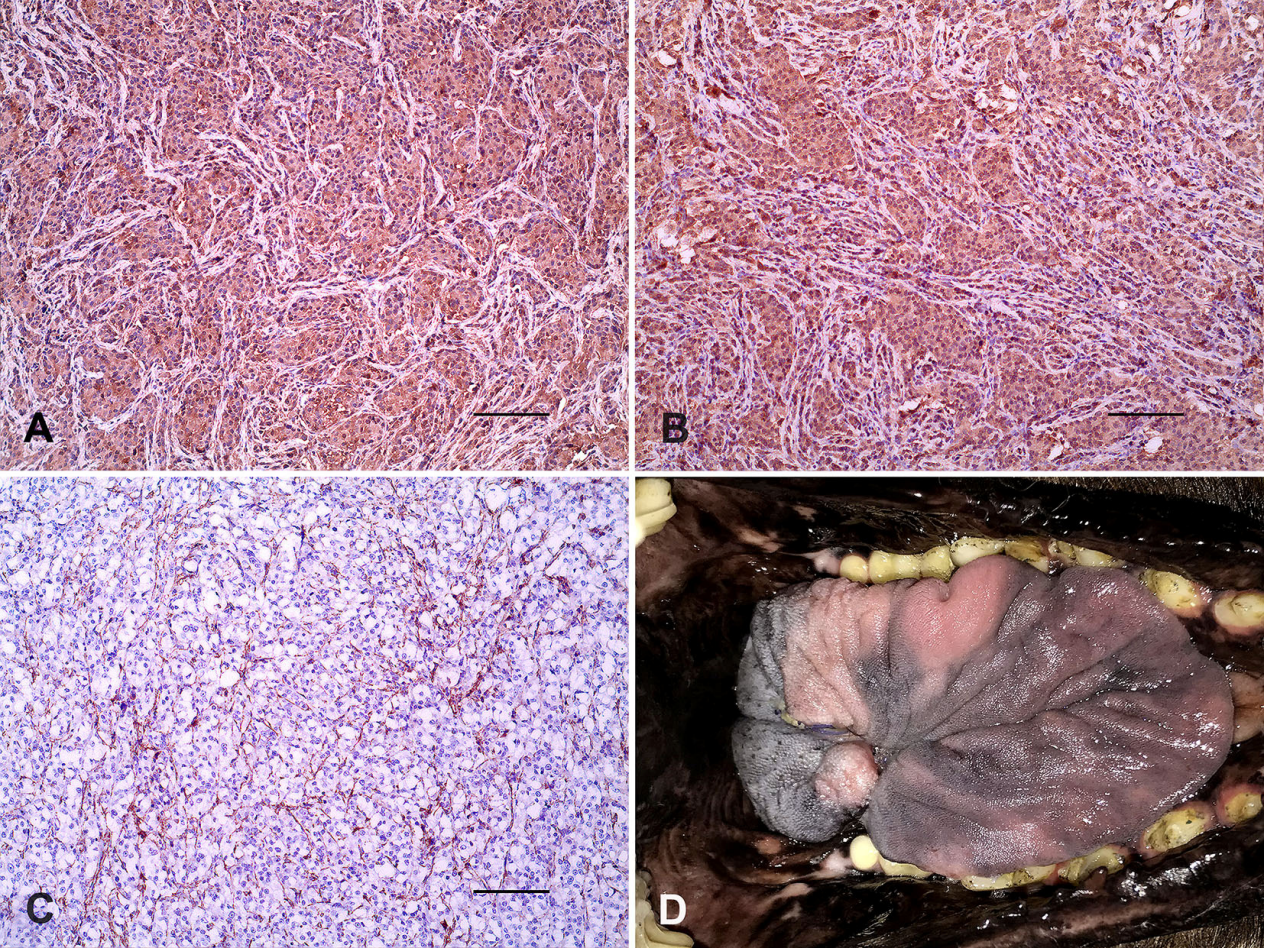
Tongue, paraganglioma. **(A)** Strong positivity of neoplastic cells for Chromogranin A. **(B)** Immunolabeling of tumoral cells for Synaptophysin. **(C)** S100 protein immunostaining of sustentacular cells. Immunoperoxidase. Bar = 100 μm. **(D)** Proper healing of the tongue 1 month after partial glossectomy.

Histologically, the lingual mass was densely cellular and composed of round to polygonal cells arranged in distinctly rounded nests surrounded by a thin fibrovascular stroma (“Zellballen” appearance) evidenced by the reticulin stain (Figure 1C). Neoplastic cells showed rounded nuclei, heterogeneous chromatin, moderate anisocytosis, and anisokaryosis, and occasional evident single nucleoli (Figure 1D). Tumoral mass also showed two mitotic figures per 10 high-power fields (2.37 mm^2^). The cytoplasm was eosinophilic and slightly granular, and PAS-negative stained. There were multifocal tumoral infiltrations to the muscular tissues and scarce surgical margins. Immunohistochemistry of tumor samples (Table 1) showed strong immunolabeling for the neuroendocrine markers Chromogranin A (Figure 2A) and Synaptophysin (Figure 2B), and vimentin, moderate GFAP positivity, and remarkable S100 protein immunostaining of sustentacular cells (Figure 2C).

The dog returned 1 month after partial glossectomy for clinical follow-up, and there was an improvement in food intake and weight gain. The tongue showed proper healing (Figure 2D), and halitosis disappeared. Post-surgical follow-up occurred for 6 months, and there was no recurrence of the lingual tumor. Morphological features and immunohistochemistry assay supported the diagnosis of primary paraganglioma of the tongue. The query performed in PubMed failed to demonstrate paragangliomas of the tongue in the Veterinary literature and a few cases in human beings. Table 2 summarizes the main features of paragangliomas of the tongue in the Chow Chow dog and human patients.

**Table 2:** General features of paragangliomas of the tongue in the Chow Chow dog and human cases.

**Features**	**Chow Chow dog**	**Human cases (14–18)**
Anatomical location	Right side of the lingual body extending to the base of the tongue	Base of the tongue (14, 16), lateral of the middle third of the tongue (15), lingual posterior aspect (17), back of the tongue squeezing the pharyngeal cavity (18).
Clinical signs	Sialorrhea, oral bleeding during eating, halitosis, and dysphagia	Paresthesia, oral bleeding during eating (14), biting on the lesion, swelling, and pain (15), chronic throat irritation (16), asymptomatic (17), impairment of pronunciation, and sleep snoring (18).
Gross findings	Ulcerated nodule	Mass connected to the epiglottis and almost obliterating the entire oropharynx (14), swelling of the tongue (15), small granulating mass (16), single lingual nodule (17, 18).
Cytology	Round to polygonal cells, occasional acinar-like structures and naked nuclei, moderate anisocytosis and anisokaryosis, stippled nuclear chromatin, and some binucleated cells	Not accomplished (14–18)
Histology	Densely cellular rounded nests (“Zellballen” appearance), thin fibrovascular trabeculae, round to polygonal cells rounded nuclei, heterogeneous chromatin, moderate anisocytosis, and anisokaryosis, eosinophilic and granular cytoplasm PAS-negative stained.	Polygonal cells grouped in nests and surrounded by a net of thin fibrovascular septae (15–18), “Zellballen” appearance (16, 17), round nuclei within fine-grained chromatin (16–18), granular eosinophilic cytoplasm (16) PAS-negative stained (15), uniform cells without atypia, mitosis, or necrosis (16, 17).
Immunohistochemistry	CrA, SYN, VIM and GFAP positivity. S100 positivity of sustentacular cells. Melan-A, WS CK and EMA negativity.	CrA and NSE (16, 17), SYN and VIM (18) positivity. S100 positivity of sustentacular cells (16–18). CK (16–18) and melanoma marker (HMB45) (18) negativity.

## Discussion

The paraganglioma of the tongue in the Chow Chow dog shares many similarities with the human counterpart. Difficulty swallowing associated with a partial tongue dysfunction owing to tumor infiltration and traumatic injuries enabled sialorrhea, difficulty feeding, bleeding, and halitosis in the dog. Hemorrhage during eating was also related to traumatic injuries of a tumoral mass on the base of the tongue in the first report of primary lingual paraganglioma in humans (14). Bleeding has not been detected in other cases of paragangliomas in humans with small neoplasms on the tongue (15–17), which possibly reduced the risk of traumatic injuries and hemorrhage.

Pharyngeal paresthesia (14), pain and swelling of the tongue (15), throat irritation (16), choke (17), and sleep snoring (18) are other clinical signs detected in human patients with paragangliomas of the tongue. Anatomical location of the tumor on the tongue possibly did not have an effect on pharyngeal areas, reducing the variability of clinical signs in the Chow Chow dog. It is also important to consider the difficulty in evaluating some clinical signs and sensations in dogs concerning paraganglioma of the tongue in comparison with their counterparts in humans.

The large ulcerated tumoral mass in the dog showed deep infiltration of the lingual muscles from the body of the tongue to the base. The high serum activity of creatine phosphokinase possibly demonstrates muscular damage related to tumor growth and infiltration. The location of paragangliomas in the tongue of humans is similar to that observed in the dog, and ulceration and serum biochemistry abnormalities have not been reported (14–17). The absence of other hematological or biochemical changes indicates the general good physical condition of the dog despite feeding difficulties and weight loss.

Cytological features of the ulcerated lingual lesion in the Chow Chow dog, such as acinar-like configuration or loose clusters of cells with round to oval nuclei within stippled chromatin, moderate anisocytosis and anisokaryosis, and some naked nuclei (19–21), provided the pre-surgical suspicion of a neuroendocrine origin tumor. Similar cytological findings were also reported in a cat with renal paraganglioma (12). The moderate cellular pleomorphism suggested a malignant neoplasm; however, the follow-up of 6 months did not detect recurrence or metastasis. An orbital paraganglioma in a dog with mild pleomorphism of neoplastic cells showed no post-surgical recurrence for 25 months (8). Most extra-adrenal paragangliomas (EPs) of dogs arise in aortic and carotid bodies, and uncommonly in other locations, with variable cytological features and cellular pleomorphism despite the grade of malignancy (5–7).

In human patients, there are different cytological presentations of paragangliomas of the head (19, 20), including similar morphological features such as observed in the dog. Cytological diagnosis of paragangliomas of the tongue has never been conducted in human patients (14–18). Cytological findings may suggest the diagnosis of paragangliomas; however, only the combination of histopathology, immunohistochemistry, radiographic studies, and tumor location is confirmatory (20, 21). Fine needle aspiration cytology of the tongue provided to be useful preoperatively in the Chow Chow dog, and should also be considered to investigate cases suspected of recurrence or metastasis.

The most relevant histological diagnostic feature detected in the tumor of the tongue was the “Zellballen” appearance composed by nests of round to polygonal neoplastic cells PAS-negative. Microscopically, tumoral infiltration to the lingual muscles was also a significant finding. Fibrovascular stroma dividing packets or nests of variable pleomorphic epithelioid to polygonal tumoral cells, and surrounded by thin trabeculae of fibrous tissue are remarkable in EPs of dogs (5, 6, 8), and have a similar “neuroendocrine packeting” pattern in horses (9, 10) and cats (11, 12).

Paragangliomas of the tongue in human patients are histologically similar to those observed in other tissues (2, 15–17, 19) and also in domestic animals (5, 6, 9–12). The low number of mitosis in the tumoral mass of the tongue was also reported in benign and malignant paragangliomas of dogs (5, 6, 8). A high mitotic index was observed in a cat with malignant renal paraganglioma (12). Criteria of malignancy based on pathological features of paragangliomas may be imprecise to assess, including mitotic index, infiltration grade, and tumor size. Distant metastasis is considered the only unmistakable finding of malignant tumors (2). Despite the absence of recurrence or metastasis for 6 months, we cannot state for sure on the absence of malignancy of the lingual tumor on the dog.

The immunolabeling for Chromogranin A, Synaptophysin, Vimentin, and GFAP antibodies is a hallmark of extra-adrenal paragangliomas in dogs and humans (2–8), and also in horses (9, 10), and cats (11, 12). Paragangliomas of the tongue in human patients have similar immunohistochemical features (16–18), as observed in the dog.

The exclusive S100 protein positivity of sustentacular cells in the Chow Chow dog was also reported in paragangliomas of the tongue in humans (16–18), and in EPs in a dog (7), and cat (12). Most EPs of dogs are anti-S100 protein negative (4, 6). Melan-A, Cytokeratin, and EMA negative immunostainings of the lingual tumoral cells were fundamental to differentiate from common oral tumors of dogs such as melanomas and carcinomas. The immunohistochemical assay showed a high similarity between the lingual paraganglioma of the Chow Chow dog and human beings (16, 17).

The tongue is an unexpected anatomical site to arise paragangliomas, a hypothesis supported by the absence of cases reported in Veterinary literature and the rarity in human beings. Once the anatomical distribution of minor paraganglia is not entirely known, paragangliomas of the tongue may arise from parasympathetic paraganglia related to the branches of facial or glossopharyngeal nerves, or in the walls of arteries (22). Although the origin of paragangliomas of the tongue is uncertain, a germline mutation in succinate dehydrogenase gene B (SDHB) highlighted a genetic basis for the development of paragangliomas in human beings (17). Canine pheochromocytomas and paragangliomas presented similar genetic alterations of SDHB and SDHD genes and other significant chromosomal changes such as the loss of chromosome 5 (23).

Granular cell tumors (GCTs) in the tongue of dogs (24–26) are one of the most challenging differential diagnoses, sharing some similar gross and microscopic features with paragangliomas (15–17). Cytologically, lingual GCTs are composed of cells with a plasmacytoid appearance and voluminous granular cytoplasm (27), which were not observed in the Chow Chow dog. Cytoplasmic PAS-negativity of tumor cells was a determinant histological feature in the differentiation between the dog's paraganglioma of the tongue and GCT (24–26). Immunostaining of neoplastic cells for Chromogranin A, such as detected in the dog, is not observed in human cases of oral granular cell tumors (28). S100 immunolabeling of tumor cells in dogs with GCTs in the tongue may be variable (24–26) and contrasts with the S100 positivity of sustentacular cells and lack of immunostaining of neoplastic cells in the lingual paraganglioma.

Despite the rarity, paragangliomas of the tongue should also be included in the differential diagnosis of the most frequent lingual neoplasms in dogs such as melanomas, carcinomas, fibrosarcomas, hemangiosarcomas, and some benign tumors such as squamous papilloma, plasma cell tumors, and GCTs. Further investigations on the anatomic distribution of paraganglia in the oral cavity, and genetic analysis of SDH genes, may contribute to the knowledge on lingual paragangliomas in dogs.

## Data Availability Statement

The raw data supporting the conclusions of this article will be made available by the authors, without undue reservation.

## Ethics Statement

Ethical review and approval was not required for the animal study because it was an expontaneous disease. Written informed consent was obtained from the owners for the participation of their animals in this study.

## Author Contributions

JS and LF performed clinical evaluations. FR, AP, DS, TW, BS-B, and MC performed pathological examinations. MC drafted the manuscript. All authors read and approved the final manuscript.

## Conflict of Interest

The authors declare that the research was conducted in the absence of any commercial or financial relationships that could be construed as a potential conflict of interest.
